# *Petrocosmea nanchuanensis* (Gesneriaceae), a new species from Chongqing, China

**DOI:** 10.3897/phytokeys.157.33625

**Published:** 2020-08-26

**Authors:** Zhi-Jing Qiu, Jun Zhang, Xavier-Ravi Baskaran, Jin Hu, Zhen-Yu Li, Zheng-Yu Liu

**Affiliations:** 1 Key Laboratory of Southern Subtropical Plant Diversity, Fairy Lake Botanical Garden, Shenzhen and Chinese Academy of Sciences, Shenzhen, 518004, China Shenzhen and Chinese Academy of Sciences Shenzhen China; 2 Chongqing Institute of Medical Plant Cultivation, Nanchuan, Chongqing, 408435, China Chongqing Institute of Medical Plant Cultivation Chongqing China; 3 Guangdong Eco-Engineering polytechnic, Guangzhou, Guangdong, 510520, China Chongqing Jinfo Mountain Forest Ecosystem Field Scientific Observation and Research Station Chongqing China; 4 State Key Laboratory of Systematic and Evolutionary Botany, Institute of Botany, Chinese Academy of Sciences, Beijing, 100093, China Sun Yat-sen University Guangzhou China; 5 Resource Center, China Academy of Chinese Medical Sciences, Beijing, 100000, China Guangdong Eco-Engineering polytechnic Guangdong China; 6 School of Life Sciences, Sun Yat-sen University, Guangzhou, 510275, China Chinese Academy of Sciences Beijing China; 7 Chongqing Jinfo Mountain Forest Ecosystem Field Scientific Observation and Research Station, Nanchuan, Chongqing, 408435, China China Academy of Chinese Medical Sciences Beijing China

**Keywords:** Gesneriaceae, new species, *Petrocosmea nanchuanensis*

## Abstract

A new species, *Petrocosmea nanchuanensis* Z.Y. Liu, Z.Y. Li & Z.J. Qiu from Mt. Jinfo at Banhe valley of Nanchuan District in Chongqing Municipality (China) is described and illustrated for the first time. Even though this new species is similar to *Petrocosmea barbata*, it has several significant morphological differences, which includes smaller leaves, repand leaf margin, densely appressed longer pubescences on both surfaces of leaves, larger flower with length of its lower lips about three times longer than that of the upper lips, oblong lower lip lobes, shorter pistil, ovate anthers and styles that are shortly pubescent or approximately glabrous above the middle. The distinct features of *P. nanchuanensis* and four relative species namely, *P. barbata*, *P. longipedicellata*, *P. cavaleriei* and *P. xanthomaculata* were also represented in depth. However, *P. nanchuanensis* is most closely related to *P. barbata*, based on molecular studies.

## Introduction

The genus *Petrocosmea* Oliver, (1887) (Family: Gesneriaceae, Subfamily: Didymocarpoideae, Tribe: Trichosporeae) was established in 1887. At present, *Petrocosmea* genus consists of 50 species classified into five sections: sect. Petrocosmea Oliv., (1919), sect. Anisochilus Hemsl., (1899), sect. Minor Zhi J. Qiu, (2015), sect. Barbata Zhi J. Qiu, (2015) and sect. Deinanthera W.T. Wang, (1985) (Oliver 1887; Wang 1985; Wang et al. 1990, 1998; Burtt 1998, 2001; Li and Wang 2005; Wei and Wen 2009; Qiu et al. 2011, 2012, 2015). Herein, we report the discovery of a new species present on Mt. Jinfo in the Banhe valley, Nanchuan District, Chongqing Municipality of China. This new species has unconstructed anthers and an abaxial corolla lip that is approximately three times longer than the adaxial one with two yellow spots at the base of the lower lip’s lobes. Hence, the new species, *P. nanchuanensis* belongs to sect. Barbata.

## Materials and methods

Measurements and observations of morphological characters of the new species, based on living individuals and specimens, were carried out in the field or greenhouse and at the herbarium. Hairs and glandular hairs and other tiny morphological characters were observed and measured by using a stereomicroscope (Nikon SMZ18). Morphological comparisons with related species were measured, based on living individuals in the greenhouse and specimens from PE, SZBG and KUN herbaria.

## Taxonomy

### Petrocosmea nanchuanensis

Taxon classificationPlantaeLamialesGesneriaceae

Z.Y. Liu, Z.Y. Li & Z.J. Qiu sp. nov.

8F24267E-FA0C-56A1-B357-61F8AFBDE16C

urn:lsid:ipni.org:names:77211190-1

Figs 1 , 2

#### Diagnosis.

*Petrocosmea nanchuanensis* is morphologically similar to *P. barbata* Craib, but is distinguished from the latter by smaller leaves, a repand leaf margin, densely appressed longer pubescences on both surfaces of its leaves, larger flower with the length of its lower lips three times longer than that of the upper lips, oblong lower lip lobes, shorter pistil, ovate anthers and styles that are shortly pubescent or approximately glabrous above the middle.

**Figure 1.**
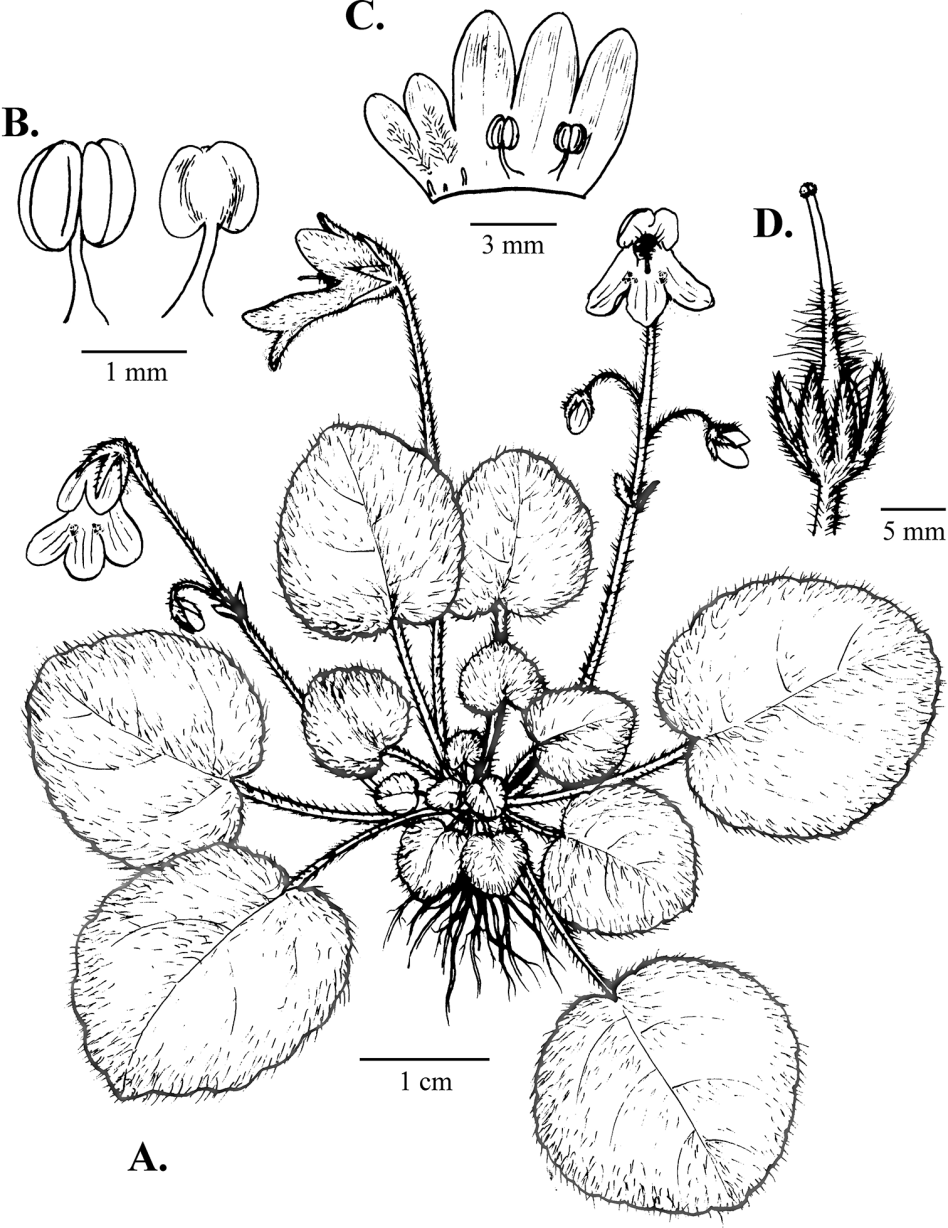
Drawing of *Petrocosmea nanchuanensis* Z.Y. Liu, Z.Y. Li & Z.J. Qiu, sp. nov. **A** habit **B** stamens **C** dissected corolla **D** calyx and pistil.

#### Type.

China. Chongqing Municipality: Nanchuan, Banhegou, Mt. Jinfo, 20 Sep 2002. *Z. Y. Li 2002016* (holotype, PE).

#### Description.

Perennial herb, rosette-forming, with a short rhizome and crowded fibrous roots. Leaves 8 to 30, all basal, crowded, the inner leaves with short or absent petioles, the outer leaves with longer petioles; leaves orbicular-ovate, broad-ovate, cordate or almost circular, 0.5–2 × 0.7–2 cm, herbaceous, apex round, base cordate, margin undulate teeth, both surfaces with densely villous, lateral veins 3 on each side, not distinct; petioles 0.5–6 cm, densely pilose. Cymes 5 to 15, 1–3 flowers per cyme; peduncle 3–10 cm, densely pilose; bracts 2 at upper- or middle-peduncle, lanceolate, 0.5–1.2 cm, pubescent, pedicel 1–5 cm, densely hairy; sepals 5-divided to the base, narrow-lanceolate, ca. 4–5 mm, pubescent externally. Corolla light purple or white, outside and inside abaxial lip puberulent, inside adaxial lip and tube near mouth densely pubescent, 2 yellow spots inside abaxial lip base; tube ca. 3 mm, adaxial lip ca. 3–3.5 mm, bi-lobed near to base, lobes ovate, abaxial lip ca. 8–9 mm, deeply tri-lobed, lobes oblong; stamens 2, ca. 2.2 mm; filaments adnate to ca. 1 mm above the base of corolla tube, ca. 1.2 mm long, glabrous; anthers ovate, ca. 1 mm long, glabrous; staminodes 3, adnate to ca. 0.2–0.4 mm above the base of the corolla tube, ca. 0.3–0.8 mm long, glabrous; pistil ca. 4.5 mm; ovary densely villous, ovoid, oblique abaxially, ca. 1.5 mm long; style unfolded pilose and glandular hairs under the middle, shortly pubescent or approximately glabrous above the middle, ca. 3 mm long.

**Figure 2.**
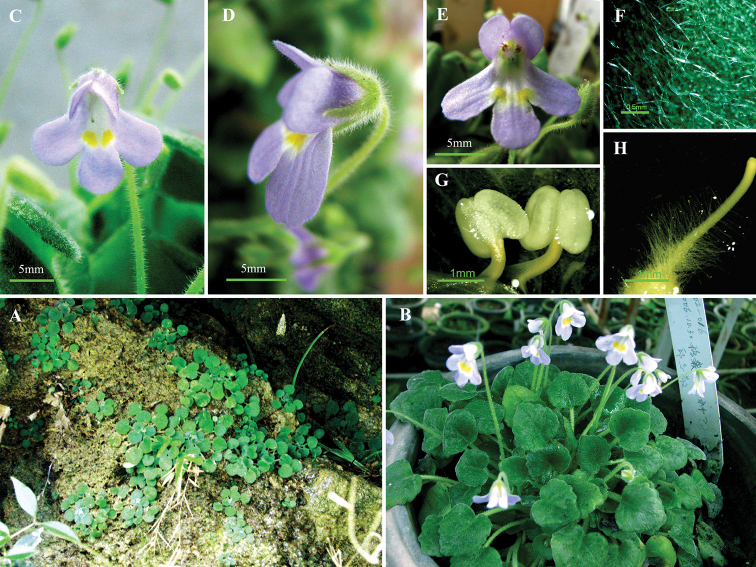
*Petrocosmea nanchuanensis* Z.Y. Liu, Z.Y. Li & Z.J. Qiu **A** habitat **B** flowering plants **C** flower, front view **D** flower, side view **E** flower, front view, showing pistil and anthers **F** hairs on abaxial leaves **G** stamens **H** hairs on the pistil.

#### Habitat and distribution.

*Petrocosmea nanchuanensis* grows on moist shady limestone cliffs or along forest edges at an altitude of 600–650 m on the Mt. Jinfo, Banhe valley, Nanchuan District, Chongqing Municipality, which is adjacent to Guizhou Province in south-western China.

#### International Union for Conservation of Nature (IUCN) Red List Category.

The new species is determined to be Critically Endangered (CR A2c) according to the IUCN Red List of Threatened Species Categories and Criteria (IUCN 2001). About 850 individuals were found in two separate and restricted populations: 300 individuals were found growing in one location with ca. 4 × 2 m^2^ and 550 individuals were found at the other location with ca. 8 × 3 m^2^. *Petrocosmea nanchuanensis* is only known from the type locality, i.e. Nanchuan District and their habitat has been threatened and damaged by deforestation and over-exploitation due to native tourism despite its protection in the Mt. Jinfo National Nature Reserve.

#### Phenology.

The new species was collected with flowers during September–November; fruits were not seen.

#### Additional specimens studied (paratypes).

China. Chongqing: Mt. Jinfo, Nanchuan, 3 Sep 2007, *R. H. Liang 2007010* (PE), *Z. J. Qiu* 2011020, QZJ-20131082 (SZG), *S.Z. Zhang* 20121077 (SZG), *Z. Y. Liu* 500119-1582ly, 500119-1595ly (IMC).

## Discussion

**Figure 3.**
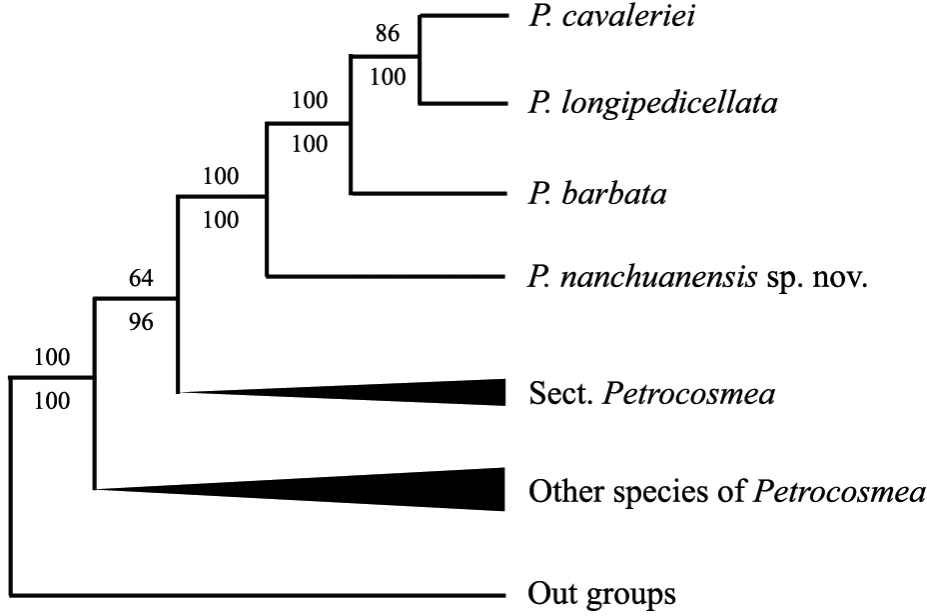
Drawing of the most parsimonious tree generated from six cpDNA and two nrDNA regions (partial & unpublished). Bootstrap values are shown above branches and Bayesian posterior probabilities are indicated below branches.

In our previous study, molecular phylogenetic analysis of *Petrocosmea* genus which includes *P. nanchuanensis* has been studied using six chloroplast DNA regions (*atpI-atpH*, *matK*, *trnH-psbA*, *rps16* intron, *trnL-F* and *trnT-L*) and two nuclear DNA regions (*ITS* and *PeCYC1D*) (Qiu et al. 2015). The molecular phylogenetic study showed that *P. nanchuanensis* rests at the base of a clade containing three other species, *P. barbata* Craib, (1919), *P. longipedicellata* W.T. Wang, (1985) and *P. cavaleriei* Levl., (1911), as shown in Fig. 3. In the phylogenetic tree, this clade, which includes four species, *P. nanchuanensis*, *P. barbata*, *P. longipedicellata* and *P. cavaleriei*, has some morphological synapomorphies, such as two yellow spots at the base of the lower lip lobes, densely villous on the ovary, villous inside corolla tube and glabrous filaments. The phylogenetic tree revealed that *P. nanchuanensis* is most closely related to *P. barbata* that also belongs to sect. Barbata.

Table 1 summarises the distinguishing features of *P. nanchuanensis* and its related species namely, *P. barbata*, *P. longipedicellata*, *P. cavaleriei* and *P. xanthomaculata* G.Q. Gou & X.Y. Wang (2010). Herein, both morphological and our previous molecular studies support *P. nanchuanensis* as a new species and being most similar to *P. barbata*. The foremost morphological differences between *P. nanchuanensis* and *P. barbata* include that *P. nanchuanensis* has smaller leaves than *P. barbata* and *P. nanchuanensis* leaves have an undulate-toothed margin, whereas, *P. barbata* has a crenate margin. Moreover, *P. nanchuanensis* shows a densely appressed villous on both surfaces of leaves instead of the adaxially puberulent and abaxial slightly and densely puberulent pattern of *P. barbata*.

**Table 1.** Distinguishing features of *P. nanchuanensis* in comparison with other related species.

Features	*P. nanchuanensis*	*P. barbata*	*P. longipedicellata*	*P. cavaleriei*	*P. xanthomaculata*
Leaf shape	Ovate	Round-ovate	Sub circular	Broadly ovate	Broadly ovate
Length of mature leaves	1.8–2 mm	2.5–2.8 mm	4–4.5 mm	2–2.5 mm	2–2.3 mm
Leaf margin	Undulate teeth	Crenate	Shallow-serrate	Repand	Repand
Hairs on the leaf	Densely villous on both sides	Sparsely appressed with pubescence above and slightly densely pubescent below	Slightly densely appressed pubescent on both sides	Pubescent on both sides	Villous on both sides
Leaf base	Cordate	Cordate	Truncate	Subcordate	Subcordate
Hairs on the peduncle and pedicel	Unfolded pilose	Sparsely pubescent and glandular hairs	Unfolded pubescent	Pubescent and glandular hairs	Unfolded pilose and glandular hairs
Length of corolla	11–12 mm	7–8 mm	8–9 mm	8–9 mm	7–8 mm
Spots inside the corolla	There are 2 yellow spots at the base of lower lip lobes	There are 2 yellow bands at the base of lower lip lobes	There are 2 yellow spots at the base of lower lip lobes	There are 2 yellow spots at the base of lower lip lobes and 3 small yellow spots in the areas of staminodes	There are 2 yellow spots at the base of lower lip lobes and 3 small yellow spots in the areas of staminodes
The degree of upper lip cracking	Near base	Near the middle	Near the middle	Near base	Near base
Do the upper lip lobes press against each other	Yes	No	No	No	Yes
Shape of upper lip lobe	Ovate	Round-ovate	Rounded	Rounded	Rounded
The degree of lower lip cracking	More than the middle	Near the middle	Near the middle	Near base	Near base
Shape of lower lip lobe	Oblong	Broadly ovate	Ovate	Oblong	Long-ovate
The length ratio of upper to lower lip	~ 1:3	~ 1:2	~ 1:2	~ 1:2	~ 1:2
Length of stamen	2–2.5 mm	3–3.5 mm	4.5–4.7 mm	1.5–1.8 mm	1.6–1.8 mm
Shape of anther	Ovate	Round-ovate	Elliptic	Oblate	Round-ovate
Length of lateral staminodes	0.6–0.8 mm	0.2–0.3 mm	0.4–0.5 mm	0.4–0.5 mm	0.4–0.5 mm
Length of pistil	4.5–5 mm	7–8 mm	6–7 mm	6–7 mm	6–7 mm
Hairs on the styles	Unfolded pilose and glandular hairs under the middle, shortly pubescent or approximately glabrous above the middle	Unfolded villous and short glandular hairs under the top, short glandular hairs on the top	Unfolded pilose under the top	Unfolded pilose and glandular hairs under the middle	Unfolded villous under the top

Likewise, *P. nanchuanensis* has larger flowers than *P. barbata*, in which *P. nanchuanensis* has flowers about three times longer in lower than in upper lips instead of about two times longer in lower than in upper lips in *P. barbata*. Additionally, lower lip lobes are oblong in *P. nanchuanensis* instead of broadly ovate in *P. barbata*, while *P. nanchuanensis* has a shorter pistil than *P. barbata*. Besides, *P. nanchuanensis* has styles that are shortly pubescent or approximately glabrous above the middle instead of styles with unfolded villous and short glandular hairs under the top and short glandular hairs on the top in *P. barbata*. The *P. nanchuanensis* has ovate instead of round-ovate anthers in *P. barbata*.

We conclude that *Petrocosmea nanchuanensis* belongs to sect. Barbata due to its floral structure, particularly anthers that are not constricted near the apex, the length of its abaxial corolla lip being twice as long as the adaxial and two yellow spots at the base of the lower lips lobes. Even though, this new species is similar to *P. barbata* and mostly varies through smaller leaves, a repand leaf margin, densely appressed longer pubescences on both surfaces of its leaves, larger flowers with their lower lips about three times longer than the upper lips, oblong lower lip lobes, shorter pistil, ovate anthers and shortly pubescent styles or approximately glabrous above the middle.

Morphological similarity between *P. nanchuanensis* and *P. barbata* has been supported by our previous molecular phylogenetic data (Qiu et al. 2015). A phylogenetic tree, based on six cpDNA regions and two nrDNA regions, confirmed that *P. nanchuanensis* is most closely related to *P. barbata* (Figure 3), which is distributed throughout Kunming County in the east-central portion of Yunnan Province, China.

## Supplementary Material

XML Treatment for Petrocosmea nanchuanensis
